# Establishment of the first bone marrow transplant program in francophone sub-Saharan Africa: clinical case and future perspectives

**DOI:** 10.3332/ecancer.2026.2100

**Published:** 2026-03-24

**Authors:** Elhadji Daouda Niang, Seynabou Fall, Sokhna Aissatou Toure, Marieme Lolita Camara, Modou Moustapha Ciss, Adjaratou Tiane Niang, Khadim Sarr, Amy Thiam, Fatou Samba Diago Ndiaye

**Affiliations:** Department of Hematology, University of Cheikh Anta Diop, Dakar, BP 5005 Dakar-Fann, BP 5005 Dakar-Fann, Senegal; a https://orcid.org/0000-0002-5113-6480

**Keywords:** Africa, transplantation, conditioning, multiple myeloma

## Abstract

**Background:**

Haematopoietic stem cell transplantation (HSCT) offers curative potential for several malignant and non-malignant hematologic disorders. Despite its proven efficacy, access to HSCT in sub-Saharan Africa remains limited, especially in francophone countries, due to the lack of infrastructure, cryobiology facilities and trained personnel. Senegal recently launched a national initiative to establish its first HSCT program.

**Case report of the first autologous stem cell transplant in Senegal:**

We report the first autologous HSCT performed in Senegal in February 2025 at Dalal Jamm University Hospital, in a 51-year-old man diagnosed with high-risk IgA-lambda multiple myeloma (ISS stage III, del17p). Mobilisation was achieved using filgrastim (10 µg/kg/day for 7 days). A total of 2.8 × 10⁶ CD34⁺ cells/kg were collected by apheresis and stored at 4°C for 24 hours without cryopreservation. Conditioning consisted of high-dose intravenous melphalan (200 mg/m^2^) followed by reinfusion of the graft on day 0. Hematologic recovery occurred by day +10, with transient grade 3 anemia and grade 4 thrombocytopenia requiring transfusion support. The main complications were manageable febrile neutropenia and mild gastrointestinal and renal toxicities. The patient was discharged on day +17, remained infection-free and achieved complete hematologic and biochemical remission at five months post-transplant. Consolidation therapy with bortezomib–thalidomide–dexamethasone and lenalidomide maintenance was subsequently administered.

**Conclusion:**

This first non-cryopreserved autologous HSCT in Senegal demonstrates the feasibility, safety and cost-effectiveness of transplantation under resource-limited conditions. Establishing local cryopreservation and molecular diagnostic capabilities will be essential to enable tandem and allogeneic HSCT, ensuring sustainability and regional self-sufficiency in advanced hematologic care.

## Introduction

The management of malignant haematological disorders remains a major challenge in most African countries, particularly in sub-Saharan Africa. This challenge is mainly due to the unavailability of diagnostic and therapeutic resources, including costly infrastructures and trained human resources with the necessary expertise. Haematopoietic stem cell transplantation (HSCT) has been a proven therapy for 6 decades in the treatment of numerous malignant haematological disorders, as well as certain benign haematological conditions and immune system diseases.

These therapies are relatively accessible in high-income countries, where the number of transplant centres continues to grow, but are far less available in low- and middle-income countries [1]. In such settings, the lack of material, human and financial resources restricts this therapeutic approach to a few specialised hospitals. Despite political will and strong commitment from medical teams, the number of transplants remains limited [2]. In Africa, where health systems are underdeveloped, only few of the 54 countries currently provide HSCT services: Morocco, Algeria, Egypt, Tunisia, Nigeria, South Africa [3], Ghana [4], Kenya [5] and Tanzania [6].

### Need for a national HSCT programme in Senegal: epidemiological rationale and public health implications

Recent epidemiological data highlight the increasing burden of haematological diseases in Senegal, both malignant and non-malignant. According to GLOBOCAN 2022, haematological malignancies represent approximately 5% of all cancers diagnosed in the country, with over 1,200 new cases each year. The 5-year prevalence of these haematological diseases is increasing: non-Hodgkin’s lymphomas (766 cases), leukaemias (323 cases), Hodgkin’s lymphomas (218 cases) and multiple myeloma (118 cases) [7].

At the same time, sickle cell disease remains the most prevalent genetic disorder in Senegal and across West Africa. It is estimated that 2% of the population are affected by a major form of the disease [8] and that around 10% of the Senegalese population carries the sickle cell trait. The disease is associated with significant morbidity due to vaso-occlusive crises, stroke and recurrent infections, with an estimated childhood mortality rate of 50%–80% in untreated severe cases.

HSCT currently represents the only curative therapy for severe sickle cell disease when an HLA-compatible donor is available [9]. However, no transplantation facility was available in Senegal prior to 2025.

The establishment of a national bone marrow transplantation programme therefore addresses a dual health imperative: curative, for malignant (myeloma, lymphomas, leukaemias) and non-malignant (sickle cell disease, aplastic anaemia) conditions; and societal, by reducing mortality and the economic burden of repeated hospitalisations and chronic treatment.

The Senegalese government has agreed to support these challenges to improve the care of patients with haematological diseases by financing the construction of a hematopoietic stem cell transplant unit within the Dalal Jamm University Hospital. This initiative is part of the national strategy to strengthen haematological and oncological care capacities, in line with the recommendations of the Worldwide Network for Blood and Marrow Transplantation (WBMT) [10]. It demonstrates Senegal’s commitment to developing a regional transplantation platform capable of meeting the needs of West African Francophone populations.

The first autologous-HSCT was successfully performed on 26 February 2025.

### Case report of the first autologous stem cell transplant in Senegal

The patient was a 51-year-old male, asthmatic, with a history of chronic epigastralgia, followed in the Clinical Hematology Department of the National Hospital Centre Dalal Jamm since 15 April 2024 for an IgA-lambda multiple myeloma, stage III according to International Staging System, with a peak in the beta zone at 72.8 mg/L at diagnosis. Cytogenetics revealed a deletion of 17p. The patient received six cycles of bortezomib (days 1, 8, 15, 22), thalidomide (100 mg daily) and dexamethasone (days 1, 8, 15, 22), with a very good partial remission at the end of treatment. He was subsequently placed on thalidomide maintenance therapy, which was stopped on 23 December 2024 in preparation for haematopoietic stem cell collection.

Before transplantation, HIV, hepatitis B and hepatitis C serologies were negative, with an anti-HBs titre of 15.25 mUI/L. The patient’s left ventricular ejection fraction was measured at 57% and the global strain was −17.4. Pulmonary function tests showed normal flow rates and Computed tomography of the chest, abdomen and pelvis was unremarkable.

Stem-cell mobilisation was achieved with filgrastim 10 µg/kg/day for seven days. Pre-collection blood count showed haemoglobin at 13.3 g/dL, marked hyperleukocytosis at 65.8 G/L with neutrophil predominance (52.16 G/L) and platelets at 187 G/L. A collection volume of 207 mL was obtained, with graft quantification showing 2.8 × 10⁶ CD34⁺ cells/kg. The CD34 stem cells counts was perfomed in the laboratory of Dalal Jamm Hospital by flow cytometry. The graft was stored without cryopreservation at 4°C for 24 hours then reinfused after conditioning with melphalan 140 mg/m^2^ IV on day −1. Reinfusion took place on 26 February 2025 (day 0) in a positive-pressure single room.

Post-transplant evolution:

- Aplasia occurred on day +5; G-CSF 5 µg/kg/day was administered until hematologic recovery on day +10 (Table 1).
- Grade 3 anemia and grade 4 thrombocytopenia required transfusion of 2 units of red cells and 35 platelet concentrates.
- The first febrile episode occurred on day 6, treated empirically with piperacillin–tazobactam + vancomycin, later supplemented by metronidazole on day 8 for persistent fever.
- Stool cultures grew *Trichomonas intestinalis*
- C-reactive protein peaked at 118 mg/L; procalcitonin was negative.
- Non-severe gastrointestinal toxicity (grade 2 nausea/vomiting), transient acute kidney injury (grade 1) and mild hypoglycemia (0.8 g/L) resolved spontaneously.

The patient was discharged home on day +17, with prophylactic amoxicillin, valaciclovir and co-trimoxazole**.**

At day +100, revaccination was initiated. At month 4, complete hematologic recovery was documented (Hb 13 g/dL, WBC 9.8 × 10⁹/L, platelets 177 × 10⁹/L). Protein electrophoresis was normal. The patient received three cycles of consolidation with bortezomib–thalidomide–dexamethasone, followed by lenalidomide maintenance. At month 5, he remained in clinical and biological remission.

### Discussion and perspectives

The first autologous-HSCT performed in Senegal represents a major milestone in the management of malignant haematological disorders. This HSCT programme is designed to improve outcomes for such patients. It is the first of its kind in francophone sub-Saharan Africa, serving as a flagship achievement for the region.

High-dose chemotherapy followed by autologous-HSCT was developed in the 1980s and has been considered the standard first-line treatment for eligible multiple myeloma patients since the 1990s [11]. Transplantation significantly improves minimal residual disease (MRD) negativity compared with chemotherapy alone (*p* < 0.001) and significantly prolongs overall survival in MRD-negative patients compared with MRD-positive ones (*p* < 0.001) [12]. Conditioning is based on melphalan followed by stem cell reinfusion [13]. Non-cryopreserved autologous HSCT has been evaluated in several low-resource countries and has shown efficacy, although haematological recovery may be slightly delayed compared with cryopreserved grafts [13, 14]. This approach avoids the need for costly cryopreservation facilities, reducing both waiting times and costs. There are additional potential benefits for patients too (such as reduced duration of hospital admission, lower rates of febrile neutropenia and mucositis [15], potential for less nausea and other Dimethyl Sulfoxide-related side effects [16, 17]. Some studies have found that non-cryopreserved stem cells are associated with faster engraftment as well (though other studies have found similar engraftment between non-cryopreserved and cryopreserved stem cells). Some studies have found that non-cryopreserved stem cells are associated with faster engraftment as well (though other studies have found similar engraftment between non-cryopreserved and cryopreserved stem cells) [15]. Beyond multiple myeloma, this approach can also be used in lymphoma. In fact, a recent meta-analysis showed that stem cell viability at transplantation was >75% in lymphomas, after a storage time of 24–144 hours at +4°C [18].

In our case, stem-cell mobilisation was successful without plerixafor, engraftment occurred by day +10 and no major complications were observed. The clinical course confirms that non-cryopreserved HSCT is feasible and safe in appropriate candidates, provided that strict aseptic and supportive measures are maintained.

According to Rajkumar [19], consolidation therapy is recommended in high-risk patients who fail to achieve complete remission after the first transplant. Given our patient’s del(17p) cytogenetic abnormality and limited access to salvage options, consolidation therapy was justified.

Ensuring access to hematopoietic stem-cell cryopreservation remains a critical challenge. Cryostorage capacity is necessary to fully implement current international recommendations, particularly for tandem transplantation and management of relapse.

As this first activity involved an autologous transplant for multiple myeloma, future steps will focus on expanding indications to other haematological malignancies, such as lymphomas and initiating allogeneic transplant programmes.

According to the WBMT recommendations for HSCT programmes in low-resource settings [10], Senegal currently meets the ideal and preferred requirements for initiating such activities. However, certain essential facilities remain lacking, including cryobiology infrastructure, molecular diagnostics (polymerase chain reaction) which is performed in private structure, nuclear imaging such as PET-scan and quality assurance systems. Sustained government support, Non-Governmental Organisation involvement and technical and financial partnerships will be necessary to ensure programme continuity.

## Conclusion

Senegal has taken a crucial step forward in haematology with the successful completion of its first bone marrow transplant. This achievement provides hope for patients in Senegal and the wider region. The first non-cryopreserved autologous transplant was carried out successfully, with five months of follow-up and no major complications. This opens the door to new prospects, including expanding transplant indications in both several malignant and non-malignant hematologic disorders and performing the first allogeneic transplant particularly in sickle cell disease, a major health concern in Senegal. Strengthening local technical capacity will be essential to support future activities.

## Conflicts of interest

The authors declare no conflicts of interest related to this work.

## Funding

The authors received no financial support for the research, authorship and/or publication of this article.

## Figures and Tables

**Table 1.** Evolution of complete blood count parameters during transplant hospitalisation.

Date	Hb (g/dL)	WBC (G/L)	Neutrophils (G/L)	Platelets (G/L)
25/02/2025	12.4	41.2	35.6	92
26/02/2025	12.4	17.55	15.9	82
27/02/2025	12.6	6.8	6.2	82
28/02/2025	12.3	1.3	1	83,6
01/03/2025	11.8	28.7	27.6	72
02/03/2025	11.5	4.7	4.5	62
03/03/2025	11.9	0.62	0.51	57
04/03/2025	11.4	0.1	0	26
05/03/2025	9	0.12	0	58,2
06/03/2025	9.9	0.17	0	35
07/03/2025	11	1.1	0.57	27
08/03/2025	11	5.1	3	10
09/03/2025	10.5	25.7	19.9	21
10/03/2025	10.8	26.4	22.5	27
11/03/2025	10.9	25.1	21.9	51
12/03/2025	12.2	9.8	5.2	97

Hb, hemoglobin count; WBC, white blood cell.
